# The use of routine outcome monitoring in child semi-residential psychiatry: predicting parents’ completion rates

**DOI:** 10.1186/s13034-015-0049-4

**Published:** 2015-06-11

**Authors:** Audri Lamers, Chijs van Nieuwenhuizen, Bart Siebelink, Thijs Blaauw, Robert Vermeiren

**Affiliations:** Curium-LUMC, Centre of Child and Youth Psychiatry, Leiden University, Endegeesterstraatweg 27, Oegstgeest, 2342 AK The Netherlands; GGzE Centre for Child and Adolescent Psychiatry, PO BOX 909 (DP 8001), Eindhoven, 5600 AX The Netherlands; Tranzo, Scientific Centre for Care and Welfare, Tilburg University, PO BOX 90153, Tilburg, 5000 LE The Netherlands

**Keywords:** Routine outcome measurement (ROM), Implementation, Youth psychiatry, Parents, Residential

## Abstract

**Background:**

Parents’ perspectives on their children’s treatment process and outcomes are valuable to treatment development and improvement. Participants’ engagement in Routine Outcome Monitoring (ROM) has, however, been difficult and may particularly be so in specialized settings, such as semi-residential psychiatry. In this paper, the use of a web-based ROM system implemented in a child semi-residential psychiatric setting is described and predictors associated with low completion rates of questionnaires by parents are identified.

**Methods:**

Parents and the multidisciplinary team of 46 children admitted to semi-residential psychiatric treatment participated in this study and completed a battery of questionnaires in three month intervals.

**Results:**

The overall completion rate of both parents during ROM assessment was 77 % compared to 83 % of all clinicians involved. Completion of questionnaires by parents was higher around first assessments and declined after a year treatment. For eight clients at least one of the parents stopped filling out questionnaires during ROM measuring. Logistic multilevel analyses revealed initial treatment factors associated with a low completion of questionnaires by parents during ROM: high comorbidity of the child on DSM Axis I, single parenthood, a higher parental educational level and having a weaker therapeutic alliance regarding goal setting.

**Conclusions:**

The findings in this paper demonstrate relatively high completion of questionnaires by clinicians and parents when using ROM in child semi-residential psychiatry. Strong administrative and electronic support undoubtedly contributed to this result. Clinicians are encouraged to motivate parents to mutually invest in ROM, and to take into account factors indicating a possible lower completion of questionnaires by parents.

## Background

In the last few years, continuous measurement of outcomes and progress in youth mental health services has received increasing emphasis [1–4]. Routine Outcome Monitoring (ROM) is the assessment of treatment outcomes at regular intervals in order to monitor clients’ progress during treatment [5]. ROM is not only an effective clinical tool for monitoring treatment outcomes at the individual level [4], it is also beneficial for research and benchmarking [6]. Although the implementation of a ROM system carries potential advantages, parents may feel that ROM adds to the burden of form-filling already required [7] and clinicians might experience ROM as increased workload [8]. Without an explicit focus on clinical use and value, ROM risks becoming just a bureaucratic burden [9] and might even negatively impact clinical interaction [7, 10]. Hence, well thought out and resourced approaches need to be developed to implement ROM in such a way that parents and clinicians experience benefits.

In spite of the growing interest in ROM, research on the actual implementation of ROM in youth mental health services is limited. Existing studies predominantly focused on aspects of the use of ROM, such as selection of ROM instruments [11, 12], the percentage of completed measures by ROM participants [1, 11, 13] and attitudes of participants towards ROM [1, 7, 10, 8]. In the youth research field, only one paper examined the effect of ROM on outcomes, showing that weekly feedback to clinicians improved youths’ symptoms and functioning [14]. This is in sharp contrast to the adult mental health field where at least 52 studies have supported the benefit of providing feedback during ROM [15]. Specific factors related to the youth mental health field complicate ROM implementation, as described by Boer and colleagues [16]. First, youth grow up; thus developmental aspects need to be taken into account when monitoring changes due to treatment. Second, youth’s problems arise during interactions with their surroundings, so assessment of youth’s functioning in several milieus deserves attention. Perhaps as a result, ROM implementation in the youth mental health field is heterogeneous regarding the number of questionnaires or assessments times used and the informants involved. A mere 16-60 % of the clinicians mentioned repeatedly using the same measurement during a clinical episode [1, 11, 13]. Additional steps in the implementation of ROM in youth mental health are needed. An important step entails developing responsive data collection systems that involve multiple informants.

Several youth studies mentioned a low completion rate of parents as an important barrier when establishing an effective ROM system [1, 2, 11]. In order to reliably monitor the effect and process of youth treatment, a high completion rate of parental questionnaires is necessary. Parents’ information, next to youth’s information, has shown to be especially valued by clinicians [2]. This is not surprisingly, since parents often have an important perspective on their children’s functioning and improvements. Further, considerable information can be gained if clinicians not only evaluate the treatment gains as perceived by the parents, but also their working alliance with parents [17]. Including parents in a ROM system could encourage them to be active participants in the care for their youth, while it also could invite them to be shared members in decision-making processes. Prior studies underlined the challenges of involving parents in ROM. One study reported that parents completed questionnaires at baseline only [1]; another mentioned that around 50 % of the parents stopped filling out questionnaires after baseline [18]. Although (government) initiatives, such as an increased support of administrative devices and implementation of electronic patient record systems, improve repeated use of measures by clinicians with 30 %; still only 6-17 % of service users complete measures repeatedly [11]. With an electronic session-by-session monitoring tool, an adherence rate was reached of 48 %, involving either a parent or youth assessment for at least two sessions [19]. A more sustained effort to involve parents in ROM is thus necessary.

Parents’ information might be especially important in psychiatric semi-residential treatment where youth with severe psychiatric disorders switch daily between home and the treatment setting. ROM research, until now, has been conducted solely in youth care residential settings or outpatient psychiatry [1]. The implementation of ROM in a semi-residential psychiatric setting is intrinsically complex due to the different treatment components provided by different team members. Nonetheless, since semi-residential psychiatric treatment is one of the most intensive forms of treatment, finding ways to improve outcomes of semi-residential treatment is required. A primary treatment goal of youth semi- residential psychiatry is re-establishing the home and school situation. Therefore, ROM could provide insight, in a standardised manner, into youth’s functioning at home and school. Furthermore, since multiple clinicians, parents and youth are involved in semi-residential treatment, a ROM system which includes mutual feedback could improve communication substantially. In addition to these clinical advantages, a ROM system could also contribute to the scarce scientific research in psychiatric (semi-)residential settings [20, 21]. Typical methodological issues for (semi-)residential settings include the lack of a control group and low response rates due to small sample sizes [22–25]. Implementation of ROM as an integral part of (semi-)residential treatment may partly overcome these limitations by providing large longitudinal datasets. Examination of factors associated with a low completion of questionnaires by participants could contribute to increased benefits of ROM for semi-residential psychiatric settings.

The aim of the present article is to describe the use of ROM implemented in child semi-residential psychiatry and to examine factors predicting completion of questionnaires by parents. A ROM system was developed, adjusted for the child semi-residential setting, and implemented in five treatment units. The emphasis when developing the ROM system was on the therapeutic relevance for the child’s treatment in terms of assessing process change together with symptom reduction. In addition, multiple informants were involved to assess these variables from different perspectives and in different milieus. Based on the earlier discrepancy between clinicians’ and parents’ completion of questionnaires in outpatient research [1, 2, 11], parents were expected to complete fewer ROM questionnaires than clinicians. As clinicians appear to especially value parents’ information during ROM [2], an effort was made to examine factors at the start of treatment in relation to completion of questionnaires by parents. Scharer’s [26] interviews with parents and clinicians in an inpatient setting revealed that the admission process is crucial for parents’ engagement during the entire treatment. Parents’ lack of participation in measurement procedures might be related to demographic variables, child’s psychiatric problems, poor parental therapeutic alliances or higher parental stress levels. Early identification of variables related to parents’ completion rates may help clinicians to support parents with mutual investment in completing ROM questionnaires.

## Methods

### Treatment setting and participants

Consecutive new admissions, 46 children ranging from 6 to 12 years in age (M = 8 years and 9 months; SD = 1 year and 6 months) at admission, to five semi-residential psychiatric treatment units between April 2011 and December 2012, were included in this study. Participants in this study were 45 mothers, 39 fathers, 2 licensed clinical psychologists, 39 teachers and 8 group workers, who each completed ROM questionnaires in three month intervals for these 46 children. The five psychiatric units were located in two cities in the western part of the Netherlands, with 26 children from location 1 and 20 children from location 2. At each location, the licensed clinical psychologist was overall responsible as a case manager for the treatment of the children. Children were admitted to semi-residential treatment for severe psychiatric problems in combination with impaired personal, family and/or school functioning. A condition for admission to semi-residential treatment was an IQ above 70. Children usually attended treatment for five days a week, for six hours a day. A multidisciplinary and tailor made approach was applied, which consisted of a therapeutic milieu on the ward, parent counselling or training, psychomotor therapy and creative therapy. Psychopharmacology was prescribed for some of the children. A highly structured therapeutic milieu is provided by group workers, who are trained in cognitive behavioural and non-violent resistance techniques to promote social-emotional competence with children. Parent counselors, most of them system therapists, conduct therapy sessions with both parents every other week. The therapy may include elements of psycho-education, parent training and system therapy. In this sample, the length of treatment differed for each child with a mean of 322 (SD = 116) days in treatment, ranging from 74 to 556 days.

### Development of a ROM system for the semi-residential setting

In the Netherlands, Boer and colleagues [16] selected psychometrically sound measures covering outcome variables most relevant for evaluating child psychiatric treatment. Two of the measures included in this package were the Dutch versions of the Strengths and Difficulties Questionnaire (SDQ) [27, 28] and the Health of the Nation Outcome Scales (HoNOSCA) [29]. Given the importance of specific parental information, especially in the child semi-residential setting, several questionnaires were added to the SDQ and HoNOSCA. These included the Dutch versions of the a) Working Alliance Inventory-revised short form (WAV-12R; [30]), b) Parenting Stress Questionnaire (PSQ) [31], and c) Family Engagement Questionnaire (FEQ) [32, 33]. In Table 1, a ROM system for the semi-residential setting is presented with measures assessing youth outcomes, treatment process and parental factors. As can be seen, multiple informants were engaged, such as group workers, teachers, case managers and mothers as well as fathers. The teacher filled in the SDQ before the start of treatment and after admission this SDQ was filled in by a group worker. Ideally, children would also be involved as informants; however, instruments need to be further developed for this purpose. With intensive administrative and electronic support, this battery of questionnaires was administered in three month intervals. The ROM questionnaires were built into a web-based computer software programme for ROM, named Patient-Reported Outcome Measurement Information System (ProMISe). The software presented each questionnaire on a separate screen with the questions and response options. After answering the last question, the person was automatically directed to the next questionnaire. Each client had an individual code and each informant had his or her own personal private secure access to the database. Furthermore, the ProMISe software helped secretaries in the management of the data collection. The secretary received automatic emails for every upcoming ‘assessment’ with detailed information about the client, assessment time and informant. Clinicians were asked by the secretary via mail to complete the questionnaires with the specific details of the child and measurement moment. After one and two weeks, a reminder message was sent by the secretary. Parents were invited to complete ROM questionnaires half an hour prior to the parent therapy session. Regular meetings between the secretary, research assistants and the helpdesk of ProMISe occurred to monitor ROM assessments.

**Table 1:** ROM design for child semi-residential psychiatry

	ROM instrument	Involved informants	Duration in minutes	Time of assessment
Child outcomes:				
Strengths and difficulties	SDQ/Parents	Fathers/ Mothers	10	Before intake, at 3 month intervals, at follow up
	SDQ/Teacher	Before intake: teacher; at three month intervals: Group Worker	10	
General functioning	HoNOSca	Case Manager	5	After the intake, with three month intervals after start of treatment
Parent process:				
Stress levels	PSQ	Fathers/ Mothers	10	Before intake, at 3 month intervals, at follow up
Parents therapeutic alliance	WAV-12R/ Parents	Fathers/ Mothers	5	After 4-6 weeks, at 3 month intervals
	WAV-12R/ Team	Case Manager	5	
Child process:				
Child alliance	FEQ	Case Manager	5	After 4-6 weeks, at 3 month intervals

*HoNOSCA* Health of the Nation outcome scales for children and adolescents, *SDQ* strengths and difficulties questionnaire, *PSQ* parenting stress questionnaire, *WAV-12R* working alliance inventory-revised

### Measures

#### DAWBA

The DAWBA (Development and Well-Being Assessment) is a web-based diagnostic interview (see www.DAWBA.com) comprising both closed- and open-ended questions designed to generate DSM-IV and ICD-10 based classifications [34]. Parents, teachers completed the DAWBA for youth. Afterwards, a clinical psychologist provided DSM classification after reviewing the symptoms, impairment and qualitative information. The initial validation study of the DAWBA showed excellent discrimination between community and clinic samples [34].

#### WAV-12R

The Dutch revised version of the Working Alliance Inventory (WAV-12R; [30]) is a 12 item questionnaire, measuring the parent-team therapeutic alliance from a multidisciplinary team’s and parents’ perspectives. The parent and team versions contain 12 slightly different items rated on a 5-point Likert scale, ranging from 1: ‘rarely or never’ to 5: ‘always’. The team version consists of three subscales ‘Bond’, ‘Goal’ and ‘Task’; Cronbach’s alpha for the total score was .94, ranging from .72 to .92 for the subscales in the current sample. The parent version consists of the subscales ‘Insight’, ‘Working’ and ‘Bond’; Cronbach’s alpha for the total score was .91, ranging from .92 to .97 for the different subscales in this sample.

#### PSQ

The PSQ is a 34-item measure assessing the parents' stress levels [31]. It yields a total parenting stress scale score as well as five sub-scores: parent–child relationship, competence, depressive moods, role restriction and physical health. A higher score indicates more experienced stress. In the current sample, Cronbach's alpha for the total score was .90, with subscales ranging from .77 to .91 for mothers. For fathers, the Cronbach’s alpha was .94 for the total score, with subscales ranging from .81 to .90.

### Additional research information

#### Sociodemographic information

Information on sociodemographics (i.e., educational level of parents, age, gender) was collected as part of a standard questionnaire in the admission process of clients to semi-residential psychiatry and compared to national data [5]. The educational level of parents was categorised according to the International Standard Classification of Education (ISCED) [5]). The ISCED classifies different types of education into nine levels, ranging from early childhood education to the doctoral level or equivalent. As a result of the small sample size, three categories were formed: early/ primary, lower/ upper-secondary and tertiary/master.

#### Informal qualitative information during implementation of ROM

Notes from monthly meetings, with the aim to evaluate ROM with all the clinicians, were collected from August 2011 until April 2013. Remarks from parents that were given to clinicians or the secretary during this period were also documented.

### Procedure

The present study, which was part of a larger study, was presented to the medical ethical board of the Leiden University Medical Centre, which considered the study falling outside the WMO (Dutch Medical Research in Human Subjects Act). Written consent was waived since all information was collected for clinical purposes. Patient data were stored anonymously in the database and data were managed in line with Dutch ethical guidelines: Personal Data Protection WGBO (Agreement on Medical Treatment Act) and WBP (Personal Data Protection Act). All clients were informed before intake that ROM is part of the general policy of Curium-LUMC to monitor treatment outcomes and will be used in an anonymous form for research purposes. For the ROM-assessment, parents needed to have sufficient command of the Dutch language. As a result, one referred client was not included in the ROM data collection as these parents indicated during the intake procedure having difficulties with the Dutch language. The aim of the larger study was to evaluate the effect of strengthening of the therapeutic alliance between parents and clinicians on treatment outcome with an A-B design. The first part (Phase A) of the sample (N = 22) received treatment as usual; the second part (Phase B; N = 24) received parent-team alliance strengthening strategies which were added to the treatment-as-usual. For this purpose, the multidisciplinary team was trained in alliance building strategies, such as promoting partnership with parents and explicitly evaluating the strength of the parent-team alliance. As these alliance building strategies could have an influence on our research question, the variable ‘treatment condition’ is included in the analyses of the current study.

### Statistical analyses

For parents together and for clinicians together (teacher, case manager and group worker), the overall completion rate of the returned questionnaires was calculated by comparing the actual number of completed questionnaires per assessment to the number of questionnaires that should have been completed during treatment per assessment for that client. In this way an overall questionnaire completion rate was generated for parents and clinicians. To examine completion rates of participants in more detail, completion rates were also calculated in the same manner, but then separately for each participant per instrument and per assessment. For descriptive analyses, SPSS (20.0) was used. Characteristics of parents were compared with national data [35] by conducting two-tailed t-tests. Based on the results of the overall completion rates of questionnaires by mothers and fathers together across all assessments, the 46 children and their parents were divided into a “high completion” group and a “low completion” group. As there are no clear guidelines in the Netherlands about what the minimal response percentage per client should be in ROM, the cut-off point was based on having a minimal of 15 clients in the smallest group. This provided the opportunity to describe demographics, youth’s psychiatric problems, parental alliance and stress at the start of treatment between the two groups. For further predictive analysis MLwiN [36] was used which implies a multilevel structure. With logistic multilevel analysis the response of both parents on each assessment as a binary dependent variable (parent did or did not complete questionnaire) was examined. The multilevel structure of analysis included assessment (level 1) grouped into individuals (level 2) grouped into mothers and fathers (level 3). Second-order PQL approximation, as implemented in MLwiN, was used. Random intercepts were allowed on the higher levels (individuals and parents); however, no random slopes were applied. Due to limited power, the analyses involved separate logistic multilevel analyses. For child-related factors, the age and treatment location were taken as covariates and for parental-related variables, treatment location and alliance intervention was considered as a covariate. Categorical variables were presented by (binary) dummy variables, which were contrasted against the base category. Multilevel analysis has the advantage of making use of all the data, although length of treatments differed between participants.

## Results

### Completion rates of questionnaires by participants during ROM

The completion rates for participants, separately, were examined in detail per assessment and per ROM instrument, as seen in Table 2. The gradual decline of the N in the upper part of the Table indicates the number of children still in treatment at each assessment, as the treatment length was variable per child. Questionnaire completion rates were higher for initial assessments and declined over treatment. Group workers show overall higher completion rates, while fathers show lower completion rates. There were nine clients with a 100 % return of completed questionnaires from all the ROM participants on all the assessments during their treatment. For six clients, one or more questionnaires from the initial assessment were missing. There were eight children of which one of the two parents stopped filling out questionnaires after the first few ROM assessments, although the treatment process continued. The mean completion rate of questionnaires, for all assessments and all instruments, of both parents per child was 77.3 % (SD = 21.9) ranging from 27.3 % to 100 %. For all clinicians (teacher, case manager and group worker) per client the completion rate for all the questionnaires was 82.6 % (SD = 15.7) ranging from 48 % to 100 %.

**Table 2:** Mean percentages of completed questionnaires of ROM participants for each instrument and each assessment

		T0 Intake (n = 46)	T1 4-6w (n = 46)	T2 3-4 m (n = 45)	T3 6-7 m (n = 39)	T4 9-10 m (n = 33)	T5 12-13 m (n = 20)	T6 15-16 m (n = 5)	FU After 1 m (n = 46)
Case M:									
	HoNOSCA	38 (83 %)		35 (78 %)	36 (92 %)	27 (82 %)	9 (45 %)	0 (0 %)	-
	WAV-12R	-	31 (67 %)	40 (89 %)	39 (97 %)	28 (85 %)	9 (45 %)	5 (100 %)	-
	FEQ	-	22 (48 %)	30 (67 %)	33 (85 %)	24 (73 %)	10 (50 %)	0 (0 %)	-
Teacher/GW									
	SDQ	33 (72 %)	-	43 (96 %)	39 (100 %)	31 (94 %)	17 (85 %)	2 (40 %)	-
Mothers									
	SDQ	43 (93 %)	-	42 (93 %)	36 (92 %)	24 (73 %)	9 (45 %)	0 (0 %)	25 (54 %)
	PSQ	43 (93 %)	-	36 (80 %)	38 (97 %)	27 (82 %)	11 (55 %)	0 (0 %)	30 (65 %)
	WAV-12R	-	37 (80 %)	39 (86 %)	38 (97 %)	25 (76 %)	17 (85 %)	5 (100 %)	-
Fathers									
	SDQ	43 (93 %)	-	32 (72 %)	32 (79 %)	23 (70 %)	9 (47 %)	0 (0 %)	23 (51 %)
	OBVL	40 (88 %)	-	33 (74 %)	33 (85 %)	27 (83 %)	8 (42 %)	0 (0 %)	22 (48 %)
	WAV-12R	-	32 (70 %)	38 (85 %)	33 (85 %)	24 (73 %)	18 (89 %)	5 (100 %)	-

Values are presented in Number of completed questionnaires and completion rate

*HoNOSCA* Health of the Nation outcome scales for children and adolescents, *SDQ* strengths and difficulties questionnaire, *PSQ* parenting stress questionnaire, *WAV-12R* working alliance inventory, *Case M* case managers, *GW* group worker, *w* weeks, *m* months, *FU* follow up one month after treatment ending

### Informal comments from ROM participants

Feedback from the participants revealed that during the implementation of the semi-residential ROM system the following aspects were appreciated: a) the feasibility of the ProMISe database and the email reminders from the secretary as mentioned by clinicians; b) the flexibility of the assessment procedures for parents as some parents preferred completing questionnaires on paper or by mail instead of electronically at the institute; and c) additional support from the helpdesk if clinicians were delayed in completing questionnaires. In addition, there were also some complaints from participants: a) the timing of the first assessment of the WAV-12R was moved six weeks later; as there was earlier not enough contact to give an adequate judgment of the alliance; b) the HoNOSCA was considered by the case managers to be aimed at adolescent problems and less suitable to address child problems; c) case managers were worried that a large number of the children diagnosed with developmental disorders would not show an improvement on the chosen questionnaires; and d) one case manager mentioned time pressure as a reason of non-completion of questionnaires. Some group workers as well as some parents expressed the wish to receive feedback. By advocating the advantages of routine measurement from different perspectives, participants were motivated to stay engaged.

### Characteristics of parents

Of the 46 children, 37 (80.4 %) lived in a two-parent home, of which 32 (69.6 %) with both biological parents, four (8.7 %) with one biological parent and a stepparent and one (2.2 %) with foster parents. Nine children (19.6 %) lived in a single-parent home, of which two (4.4 %) lived in two single-parent homes (divorced parents with shared custody). No significant difference emerged between this sample regarding family composition and national data (*p* = 0.14); national data showed that 13 % of Dutch children lived in a single-parent home. Parents’ educational level was 2.3 % for mothers and 2.6 % for fathers early/ primary level (national data: 8.4 %), 77.3 % mothers and 68.4 % fathers lower/upper-secondary level (national data: 63.1 %) and 20.4 % mothers and 29 % fathers tertiary/master level (national data: 27.6 %). This educational level was: a) fairly similar to national data, b) not significantly different between fathers and mothers, and c) equal between both treatment locations. Forty-four children (95.7 %) had two Dutch parents. One child (2.2 %) had one Dutch and one non-Dutch parent and one child (2.2 %) had two non-Dutch parents. These groups were much smaller than in national data (*p* = .00), in which 9 % of the children had one non-Dutch parent and 14.4 % of the children had two non-Dutch parents. Overall, there were no significant differences between the two treatment locations or the two treatment conditions (alliance strengthening strategies) with regard to the baseline sociodemographic variables thus warranting combining the whole sample in further analyses.

### Description of a low and high questionnaire-completing group of parents

**Fig. 1:**
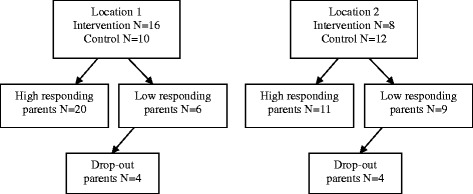
Flowchart of parents’ responsiveness regarding questionnaire completion during Routine Outcome Monitoring

To provide an opportunity to describe “high completion” and “low completion” of parents, a cut off point was chosen at an overall completion rate of 70 %. This completion rate is based on the overall completion rates of both mothers and fathers, for each child separately, on all the assessment times. The result is two groups of clients of who parents show “low” (n = 15) and “high” (n = 31) questionnaire-completion. Of the 15 clients in the low completion group, six were at treatment location 1 and nine were at treatment location 2. Figure 1 shows the participation of parents in ROM assessments at different stages of the study. There was approximately the same number of low completion parents in the alliance strengthening group as in the treatment as usual group. In both groups there was one client with parents from a non-Dutch background. The characteristics of both groups are shown in Table 3. The low completion group involved more single parents and more children with comorbidity on Axis I in the DSM-IV classification. Also, there was more stress related to physical health problems for mothers in the low completion group. Case managers tended to experience lower therapeutic alliance with respect to the agreement made with parents regarding tasks and goals in the low completion group.

**Table 3:** Characteristics of the 46 children and their families between a high and low questionnaire completion group of parents

Variables at baseline		High Completion		Low Completion	
		(n = 31)		(n = 15)	
Age		9.2(1.5)		8.3(1.6)	
Female %		22.6		13.3	
Family composition					
	Biological parents %	77.4		53.3	
	Single parents %	9.7		40	
	Other %	12.9		6.7	
Parental educational level					
	Early/ primary %	3.2 ^a^	0^b^	0^a^	7.1^b^
	Lower/ upper secondary %	77.4^a^	71^b^	66.7^a^	28.6^b^
	Bachelor/ master/ doctoral %	19.4^a^	19.4^b^	20^a^	35.7^b^
Days in day treatment		324		318.6	
DSM-IV AXIS I classification					
	PDD %	77.4		53.3	
	ADHD/ODD %	6.5		6.7	
	Mood and anxiety disorders %	6.5		13.3	
	Other disorders %	9.7		29.7	
Presence comorbidity on DSM-IVAXIS I %		38.7		60	
Parenting stress level					
	Parent–child relation	12(3.0)^a^	10.9(3.3)^b^	11.7(4.2)^a^	10.5(4.3)^b^
	Parenting	15.5(3.1)^a^	15.1(3.2)^b^	15(3.9)^a^	15.5(6.5)^b^
	Depressive mood	11.2(2.8)^a^	10.7(2.9)^b^	11.8(2.5)^a^	10.5(3.2)^b^
	Role restriction	13.1(5.9)^a^	10.3(3.9)^b^	12.6(4.6)^a^	10.4(4.9)^b^
	Physical health	12.7(4.2)^a^	10.6(3.1)^b^	15.9(4.0)^a^	11.3(4.5)^b^
Parent-team alliance					
	Bond	13.5^c^		13^c^	
	Task	14^c^		12.9^c^	
	Goal	14.9^c^		12.7^c^	
	Insight	5.7^a^	6.1^b^	5.3^a^	5.5^b^
	Bond	15.0^a^	14.9^b^	14.6^a^	15.2^b^
	Task/Goal	20.0^a^	21.5^b^	21.8^a^	21.4^b^
Treatment condition: alliance strengthening %		54.8		46.7	

Values given are means (SD), unless otherwise indicated

Higher scores reflected higher stress level/ more symptoms/stronger alliance

*PDD* pervasive development disorder, *ADHD/ODD* attention deficit/hyperactivity disorder/oppositional defiant disorder

^a^From the perspective of mothers

^b^From the perspective of fathers

^c^From the perspective of casemanagers

### Predicting parents’ completion of questionnaires during ROM in a semi-residential setting

Separate logistic multilevel analyses were conducted with the response of parents on each assessment as binary variable and the results are presented in Table 4. A low completion of questionnaires by parents on the ROM data collection system was significantly predicted by parent related variables as well as a child related variable. Odds Ratios of significant parent variables are: single parenthood .39 (*p* = .01), a higher parental educational level .44 (*p* = .01) and a weaker therapeutic alliance between the team and parents on goal setting 1.39 (*p* = .00). Stress of mothers related to physical health .94 (*p* = .05) was close to being a significant variable. A child related variable with a significant Odds ratio turned out to be ‘high comorbidity on DSM-IV Axis I (.46; *p* = .00).

**Table 4:** Logistic multilevel analyses with parents’ completion of questionnaires over time as binary dependent variable

Predictor			Odds Ratio (OR)		(95 % CI)		*p*	
Comorbidity child^ab^			.46		.33-.76		**.00**	
Single parents^a^			.39		.19-.83		**.01**	
Parent educational level			.44		.23-.84		**.01**	
Parental alliance^ac^								
	Insight^d^	Task^e^	.92^d^	1.09^e^	.79-1.08^d^	.85-1.40^e^	.31^d^	.85^e^
	Bond^d^	Bond^e^	1.00^d^	.98^e^	.93-1.06^d^	.76-1.26^e^	.88^d^	.96^e^
	Task/Goal^d^	Goal^e^	1.06^d^	1.39^e^	.97-1.17^d^	1.12-1.73^e^	.21^d^	**.00** ^**e**^
	Total alliance score		1.00^d^	1.08^e^	.97-1.04^d^	.98-1.19^e^	.87^d^	.14^e^
Parental stress^ac^								
	Parent–child relation		1.02		.94-1.11		.68	
	Parenting		1.02		.95-1.11		..58	
	Depressive mood		.97		.87-1.08		.55	
	Role restriction		1.00		.95-1.06		.89	
	Physical health		.94		.89-1.00		.05	
	Total stress score		1.00		.98-1.02		.69	
Alliance Intervention			.70		.39-1.24		.22	

Each predictor was employed in a separate multilevel analysis

*p* ≤ 0.05(bolded)

^a^Controlling for treatment location

^b^Controlling for age

^c^Controlling for Alliance Intervention

^d^From the perspective of parents

^e^From the perspective of case manager

## Discussion

The implementation of ROM is widely recognised as being difficult, though important for improving treatment effectiveness in youth care. One important hindrance is the poor completion of questionnaires by parents, particularly at re-assessment. The present study contributes to the implementation of ROM in youth psychiatry by: a) describing the implementation of a ROM system in a child semi-residential setting and b) identifying factors associated with a low completion of questionnaires by parents. The implementation of a ROM system in a semi-residential setting of a Centre for Child Psychiatry resulted in a considerably high completion of questionnaires by clinicians (83 %) and parents (77 %). For 20 % of the clients, there was a 100 % return of questionnaires from all the ROM participants (parents, clinical psychologist, former teachers and group workers) at all the three month interval assessments. As expected and in line with earlier research, the completion of questionnaires by parents was somewhat lower than the completion by clinicians. The perspective of parents is important to researchers and clinicians and may even be more so in semi-residential psychiatry as children switch daily between home and the treatment unit. Therefore, the current study focused in detail on factors associated with parents’ completion of questionnaires. Being a single parent, a higher educational level of parents, a weaker therapeutic alliance between the team and parents on goal setting and more comorbidity on DSM-IV (AXIS I) of the child were factors associated with a low completion of questionnaires by parents to ROM.

Whereas previous research reported challenges to engage parents in ROM assessments [11], in this study three-quarters of the parents filled out the questionnaires repeatedly. One factor that might have contributed to the high completion rates of parents (77 %) and clinicians (83 %) in our study is the growing positive attitude of participants towards regular monitoring of treatment outcomes and process. In the Netherlands, ROM is being given substantial attention in order to create transparency in the effectiveness of treatments. A recent qualitative process evaluation of ROM indicated that team members, administrative staff, young people and their parents/carers supported regular monitoring of outcome if the system was easy to implement [37]. The implementation strategy used in this study might have contributed to the high completion rates, for example extra motivating phone calls to parents were made by the secretary and the helpdesk provided support to clinicians. Clinicians mentioned the feasibility of the ProMISe database, appreciated the email reminders from secretary and the helpdesk support. The strong engagement from the administrative staff and research assistants undoubtedly helped in making sure the questionnaires were completed on time. Our findings are in line with research of Hall and colleagues (2014), which showed a successful enhancement of clinicians’ completion rates with 30 % (2014) and found a completion rate for families of 49 % after implementing an electronic session by session monitoring tool. Our study shows that enhancing questionnaire completion rates is not only possible for clinicians; with a strong effort parents can show a high engagement in ROM.

Notwithstanding these high completion rates, for 17 % of the clients one of the two parents stopped with ROM assessments after questionnaire completion during the first few assessments. Completion of questionnaires was higher at the first few assessments, however, after one year of treatment declined. This ROM drop-out was, however, small compared to Van Sonsbeek and colleagues [18] who experienced 50 % drop-out after the baseline assessment. In order for a ROM system to be beneficial and useful, it must provide information that clinicians need and are willing to use [17]. Clinicians especially value the perspective of parents on the youth’s functioning during treatment [17]. Therefore, it is of interest to examine factors predicting parents’ completion of questionnaires during ROM assessments. Multilevel analysis resulted in initial variables at the start of treatment that predict completion of questionnaires by both parents. Remarkably, more comorbidity on AXIS 1 of the DSM-IV was a significant predictor of lower completion of questionnaires by parents. A possible explanation might be that children with more comorbidity, show more psychiatric symptoms, which puts more pressure on parents. Single parenthood also showed to be a significant predictor of a low completion of questionnaires by parents. Single parents are likely to be more occupied with tasks related to the care of a child with psychiatric problems as compared to those supported by a partner. As a result, the timely completion of questionnaires might be a challenge. Higher educational level of parents, especially of fathers, turned out to be another predictor. Fathers with a higher educational level might be more occupied by work. In addition, a weaker alliance as rated by the case manager regarding goal setting at the beginning of treatment was identified as a significant predictive variable. Apparently even at the beginning of treatment, it was more difficult for case managers to set mutual treatment goals with the parents who have difficulties completing questionnaires during the treatment of their youth. This finding is in line with prior research showing the impact of a strong early parental alliance on treatment attendance [38]. Remarkably, the extra investment of team members in alliance building strategies didn’t seem to influence completion rates of parents during ROM. Extra attention from clinicians is needed at the beginning of treatment for problems regarding mutual goal setting. Clinicians mentioned in interviews that ROM with feedback could be especially beneficial for clear goal setting and evaluation [8]. Last, parents’ stress related to physical health was close to being a significant predictor. From the descriptive analyses it can be delineated that mothers in the low completion group experience more physical health related stress. Mothers experiencing more physical health related stress might be less capable in finding time to complete questionnaires.

The findings need to be considered in light of the small sample size due to the specialised setting. Sample size limitations can have implications for the significance and the generalizability of the results. For example, caution is needed when generalizing these findings to other clinical settings in the youth mental health field. Strength of this study, however, is that this is the first study to use ROM in such a specialised psychiatric setting and that longitudinal assessment with three month intervals was conducted. Next, descriptive findings might have been influenced by the choice of a relatively arbitrary cut-off point to divide the group of parents into low and high responders. There are no clear guidelines in the Netherlands about what the minimal completion rates per client should be in order for ROM to be beneficial. However, the subsequent use of multilevel analyses, with the completion per assessment of both parents as a binary variable, strengthened the statistical analyses. In addition, although multiple perspectives on outcomes as well as process factors during ROM were included, the youth’s perspectives were not assessed. It remains a task for future research to develop routine instruments that could also be administered to youth. Last, the questionnaires did not allow additional comments, although parents mentioned during interviews the value of adding their own comments during ROM [10].

This study can be regarded as an important first step in demonstrating potential benefits of a ROM system for a child semi-residential psychiatric setting. The implementation strategy chosen in this study involved a relative small pilot project with five multidisciplinary teams and 46 clients only. A consequence of such a small project was more attention could be given to every individual participant than if implemented on a larger scale. It could be reasoned that the project size contributed to the difference between the completion rates mentioned in this study and the completion rates reported in prior studies. However, it has been argued that ROM implementation is more likely to succeed if started with small pilot projects that can later be extended and refined, rather than attempts to implement across a whole service [39]. To avoid wasting effort and “the goodwill” of clinicians and clients, careful approaches to ROM implementation are needed. Due to the complex setting of child semi-residential psychiatry, a comprehensive battery of questionnaires was implemented involving multiple informants and assessments in three months intervals. Remarkably, despite this considerable effort asked from clinicians and parents, this ROM system for the semi-residential setting appeared feasible to use.

Clearly, the next step would be to implement this ROM system in a semi-residential setting with feedback to the participants as an integral part of routine clinical practice. Bickman and colleagues [14] found in their Randomized Controlled Trial that routine measurement and feedback improved outcomes with youth in home-based mental health treatment in community settings. ROM feedback has been considered to improve communication, share decisions between the multiple participants and contribute to stronger therapeutic alliances [40, 41]. An electronic administered session-by session monitoring with direct feedback showed a stronger engagement from youth [19]. One can imagine that the completion of questionnaires by parents increases when they receive feedback on the completed questionnaires from clinicians.

## Conclusions

In this paper, collecting ROM information from more than one participant, especially from parents in complex youth psychiatric treatment settings is advocated. Findings may facilitate early identification of parents at risk of dropping out of a residential ROM system. A high completion of questionnaires by parents is needed to: a) make feedback during ROM data collection a useful clinical tool and b) collect large longitudinal datasets to conduct methodologically sound research. Whether a low completion of questionnaires is an indication of suboptimal treatment motivation and worse outcomes should be studied in the future. In line with the recommendations of Moran and colleagues [10], ROM should become a collaborative and meaningful process in partnership between clinicians and parents in order to improve the process and outcome of treatment for youth. ROM implementation in specialized youth psychiatric services needs further improvement in the right direction.
